# Editorial: Autonomous magnetic resonance imaging

**DOI:** 10.3389/fnimg.2023.1277580

**Published:** 2023-08-29

**Authors:** Sairam Geethanath, Rita G. Nunes, Jon-Fredrik Nielsen

**Affiliations:** ^1^Accessible Magnetic Resonance Imaging Laboratory, Icahn School of Medicine at Mt. Sinai, New York, NY, United States; ^2^Department of Bioengineering, Institute for Systems and Robotics–Lisboa, Instituto Superior Técnico, Universidade de Lisboa, Lisbon, Portugal; ^3^Department of Radiology, University of Michigan, Ann Arbor, MI, United States

**Keywords:** automation, neuroimaging, deep learning, accessible magnetic resonance imaging, accelerated magnetic resonance imaging

Magnetic Resonance Imaging (MRI) has significantly advanced our understanding of the human brain in health and disease. However, two-thirds of the world does not have access to MRI (World Health Organization, 2017; Geethanath and Vaughan, 2019). A key contributor to this inaccessibility is the absence of skilled human resources required to operate MRI machines in these regions, characterized by large populations and low resources. In developed countries, inefficient workflows result in financial burden and reduced temporal access (van Beek et al., 2019). These challenges necessitate augmenting human expertise through automation to improve efficiency across the neuroimaging pipeline. A preliminary implementation by Ravi and Geethanath (2020) outlined the scope and framework for such automation, followed by a review of developing and deploying deep learning models (DL) for MR-based neuroimaging (Aggarwal et al., 2023). This Research Topic comprises one review, one clinical trial, and six research articles demonstrating the use of DL models and feature engineering to automate brain MRI exams to reduce acquisition time, improve segmentation accuracy, and reduce contrast agent dosage.

Moya-Saez et al. review the contributions of DL to reduce Gadolinium-based MR contrast dosage through synthetic contrasts. The authors describe the different data types, neural network architectures, and assessment metrics associated with DL approaches. This article's limitations and future trends sections serve as a well-balanced guide for readers interested in pursuing research in this area. In particular, the review finds that DL-based synthesis of contrast-enhanced MR images can positively impact clinical studies by reducing patient discomfort, acquisition time, imaging cost, and, most importantly, alleviating safety concerns related to Gadolinium-based MR contrast usage. The review suggests that a combination of synthetic and quantitative MRI methods can accelerate image acquisition, automated diagnosis, and prognosis in neuro-oncology.

Given the wide variety of available protocols and applications, the need to automate MR data acquisition stems from the complexity of choosing and optimizing the proper pulse sequences for the appropriate neuroradiological investigation. Hoinkiss et al. address this contrast selection and optimization question by developing machine learning-based domain-specific language (DSL) to describe and formulate clinical demands resulting in an optimized pulse sequence for that specific request. Their simulated and acquired MR data show high correspondence to the requests, such as optimizing for contrast, signal-to-noise ratio (SNR), motion insensitivity, and geometric distortion reduction made using their DSL method.

Another approach to accelerating MR data acquisition using intelligent protocolling and subject-specific image denoising is the focus of the research article by Ravi et al.. The role of MRI in imaging Alzheimer's disease (AD) patients is well-established (Jack et al., 2008). However, the authors note that these protocols need skilled personnel to optimize acquisition parameters for each pulse sequence. The authors demonstrate speeding up a brain screen protocol using a look-up-table and search method called “intelligent protocolling” to scan vast parameter ranges typically impossible by a single MR scanner operator. This approach is compared with a vendor-supplied gold standard protocol and a human expert-produced express protocol for savings in time, MR contrast, and SNR. The approach provides a trade-off between SNR and acquisition time. The reduction in SNR is mitigated by DL-based denoising that is contrast and subject-specific. The researchers claim a gain of 1.94 × in acceleration while maintaining similar standards of contrast and SNR. They also investigated the effect of their approach on automated brain tissue segmentation and volumetry, given its significance in AD imaging, and found excellent agreement in twenty-seven locations.

Rao et al. focus on improving the accuracy of automated T1w brain segmentation by combining convolutional neural networks and transformers. They leverage the generalizability features of transformers in image-processing tasks to ensure that their algorithm performs robustly across multiple T1w datasets acquired on different scanner platforms, field strengths, neuropsychiatric conditions, and acquisition settings. Their model achieved the best segmentation and generalization performance compared to benchmarks across four multi-site datasets. You and Reyes investigate another challenge related to brain tumor segmentation: the effect of contrast and texture modification on the accuracy and generalization of brain tissue segmentation. In this work, they assess large-scale datasets and simulated MR protocols to explain model performance variations due to contrast and texture modifications. The results confirm the prior understanding that these modifications improve the accuracy of the models and further identify the improved or worsened regimes of model performance for different acquisition parameters. Finally, they note a spatial attention shift in the trained models corresponding to the applied modifications. These insights enable implementers to consider the effect of acquisition parameters, contrast, and texture modifications on their model's performance in their studies.

Another study by Lei et al. focuses on feature engineering using the least absolute shrinkage and selection operator (LASSO) to classify features into high- and low-information groups. The authors use this grouping strategy to effectively use radiomics data to grade hepatocellular carcinoma (HCC) using a dictionary of high-information features. HCC is the most common form of liver cancer, accounting for 90% of the cases. The authors demonstrate that their method improves HCC grading accuracy and performs similarly to state-of-the-art methods. Feature selection is also an active study area in ischemic stroke research (Dragoş et al., 2023). Chen and Li investigate the ability of brain MRI-derived dynamic amplitude of low-frequency fluctuation (dALFF) to discriminate between patients with acute basal ganglia ischemic stroke (BGIS) with motor dysfunction and healthy controls. They observed abnormal dALFF in BGIS patients and its variability as a potential tool to assess motor function in such patients. Ji et al. combine dALFF and static ALFF (sALFF) neuromarkers to distinguish minimal hepatic encephalopathy (MHE) patients from cirrhosis patients. Further, they demonstrate the ability of dALFF to predict the severity of liver damage in MHE patients.

This Research Topic of articles disseminates the diverse state-of-the-art automation approaches applied to multiple neuroimaging investigations. Following and implementing the US Food and Drug Administration's guidance on good machine learning practices (Harvey and Gowda, 2020) while advancing these studies, such as including explainable AI, is critical for the widespread adoption of these innovations.

## Author contributions

SG: Conceptualization, Writing—original draft, Writing—review and editing. RN: Writing—review and editing. J-FN: Writing—review and editing.
